# Supplementary figures and images for: Modeling CRISPR gene drives for suppression of invasive rodents using a supervised machine learning framework
Source: PLoS Comput Biol. 2021 Dec 29;17(12):e1009660. doi: 10.1371/journal.pcbi.1009660 (PMC8716047; doi:10.1371/journal.pcbi.1009660)

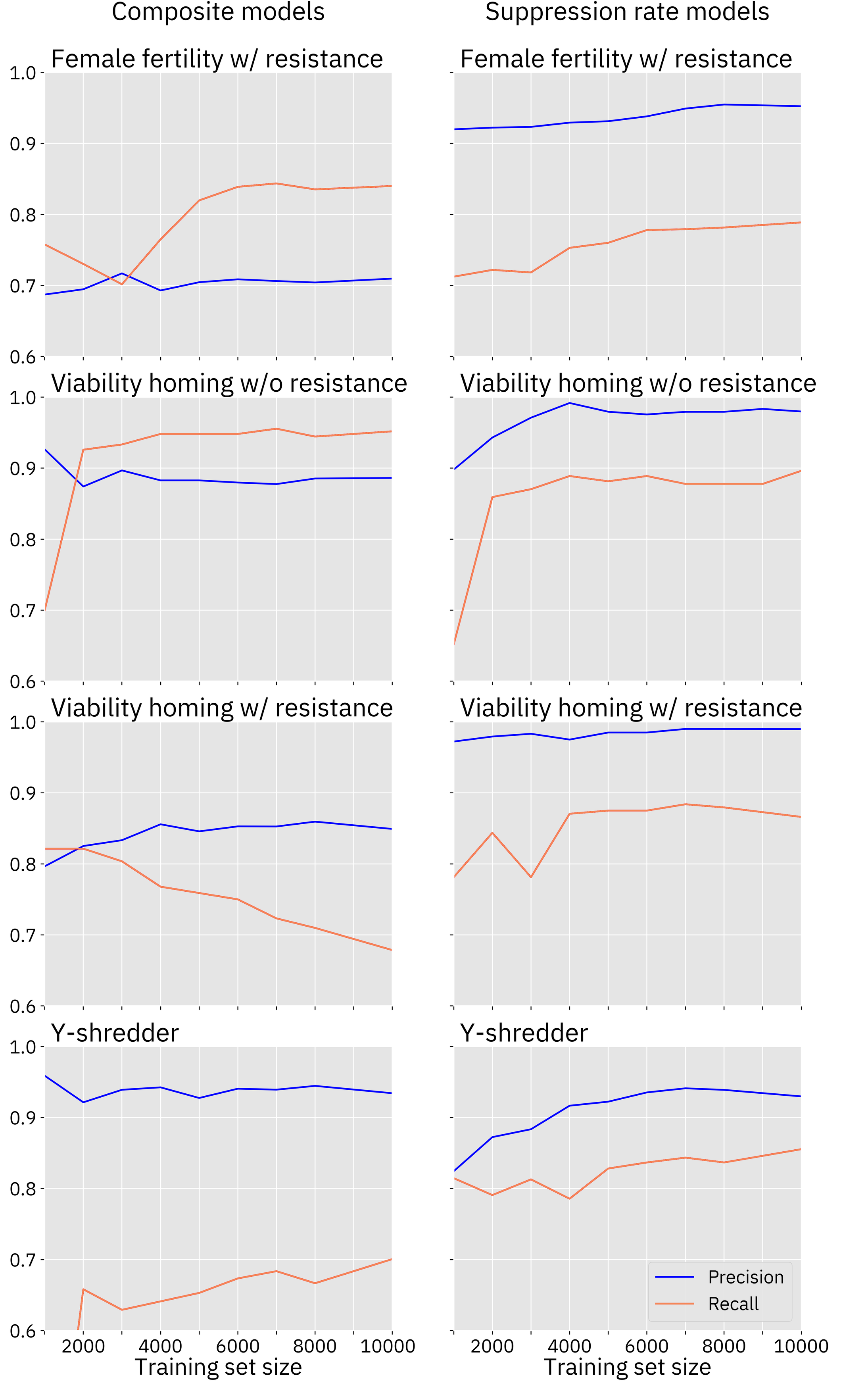

Supplement: S1 Fig — Precision and recall are shown for each of the Gaussian process models used in this study. Each model was evaluated against the Latin Hypercube test set prepared from the modeled drive. (TIF) [file pcbi.1009660.s001.tif]

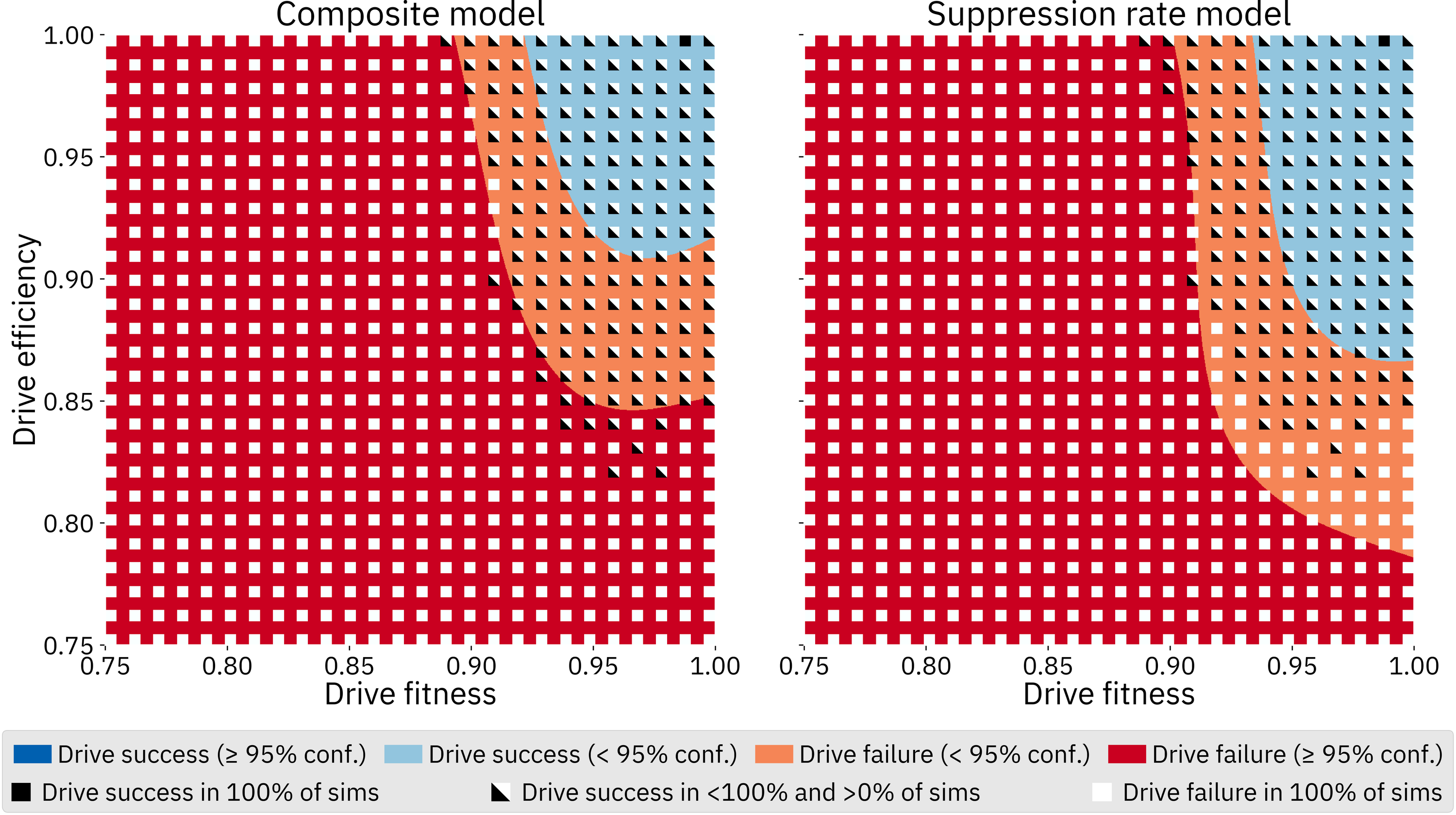

Supplement: S2 Fig — Resistance was set to 0.01 and relative R1 resistance rate was set to 0.001. Other parameters are fixed at default values. Black, gray, or white square dots show the results from actual simulations, each denoting the result of twenty simulations. (TIF) [file pcbi.1009660.s002.tif]

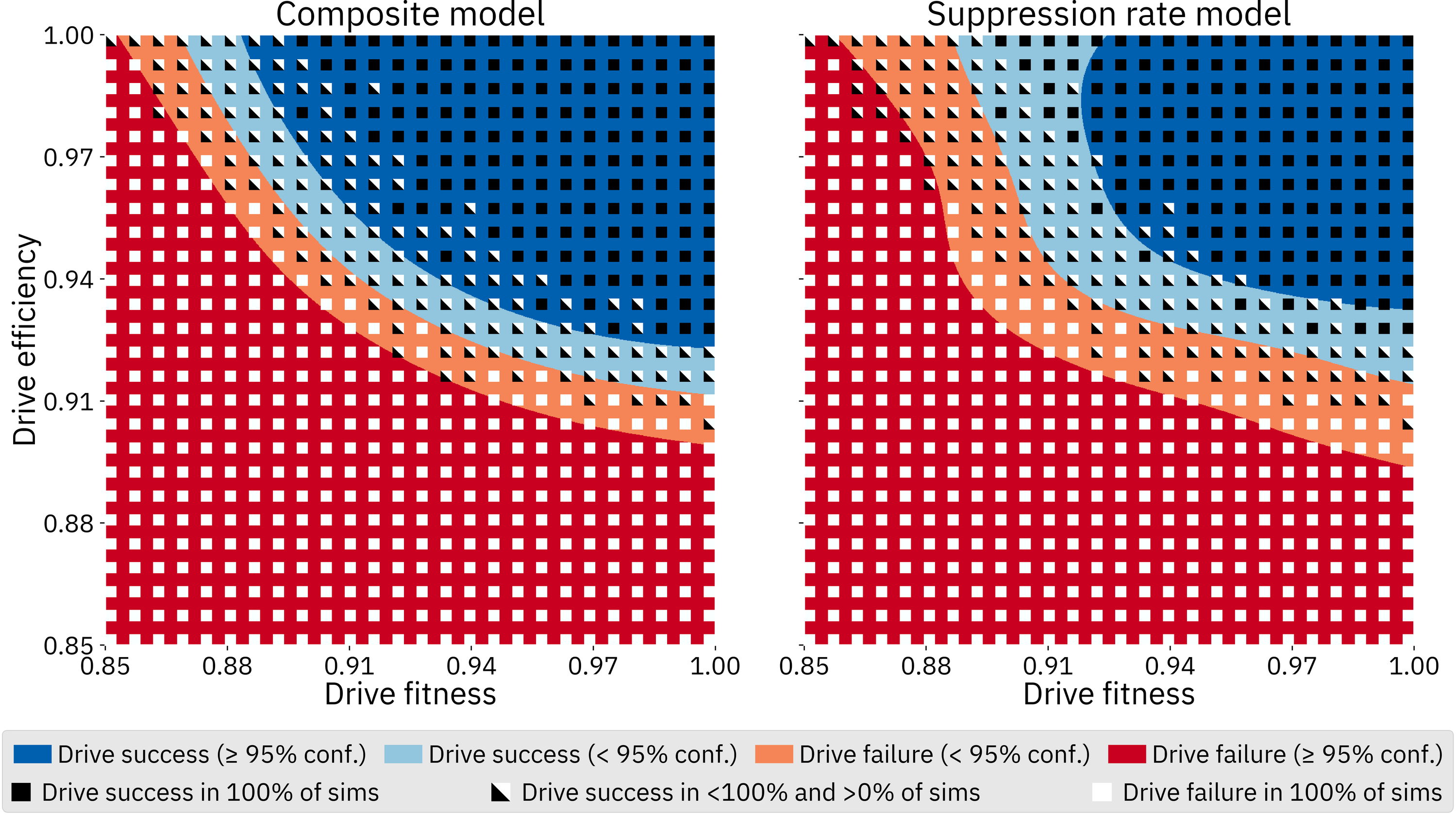

Supplement: S3 Fig — Survival rate was set to 0.8, and other parameters were fixed at default values. Black, gray, or white square dots show the results from actual simulations, each denoting the result of twenty simulations. (TIF) [file pcbi.1009660.s003.tif]

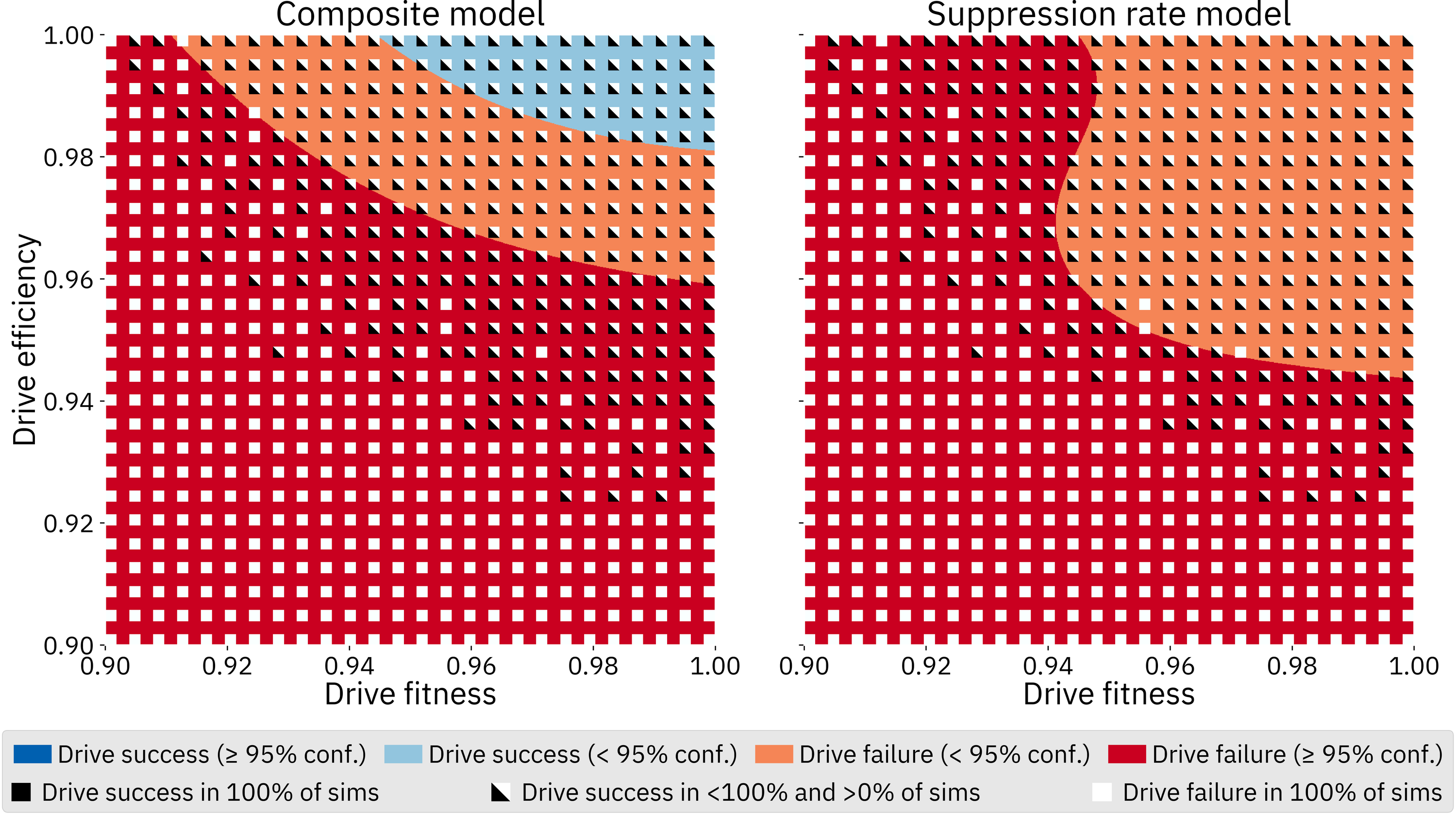

Supplement: S4 Fig — Resistance was set to 0.01, relative R1 resistance rate was set to 0.001, and survival rate was set to 0.8. Other parameters were fixed at default values. Black, gray, or white square dots show the results from actual simulations, each denoting the result of twenty simulations. (TIF) [file pcbi.1009660.s004.tif]

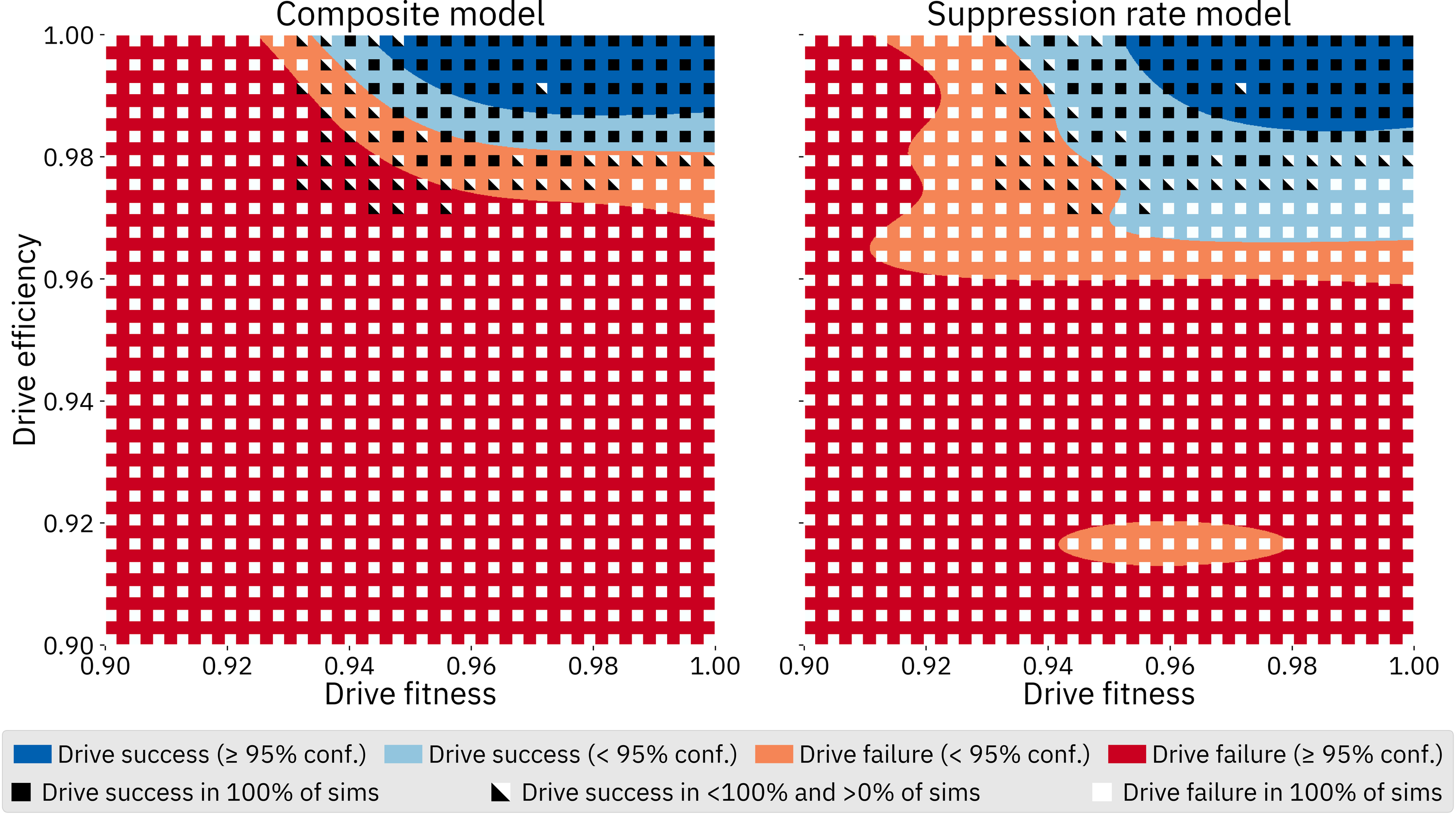

Supplement: S5 Fig — Survival rate was set to 0.8, and other parameters were fixed at default values. Black, gray, or white square dots show the results from actual simulations, each denoting the result of twenty simulations. (TIF) [file pcbi.1009660.s005.tif]

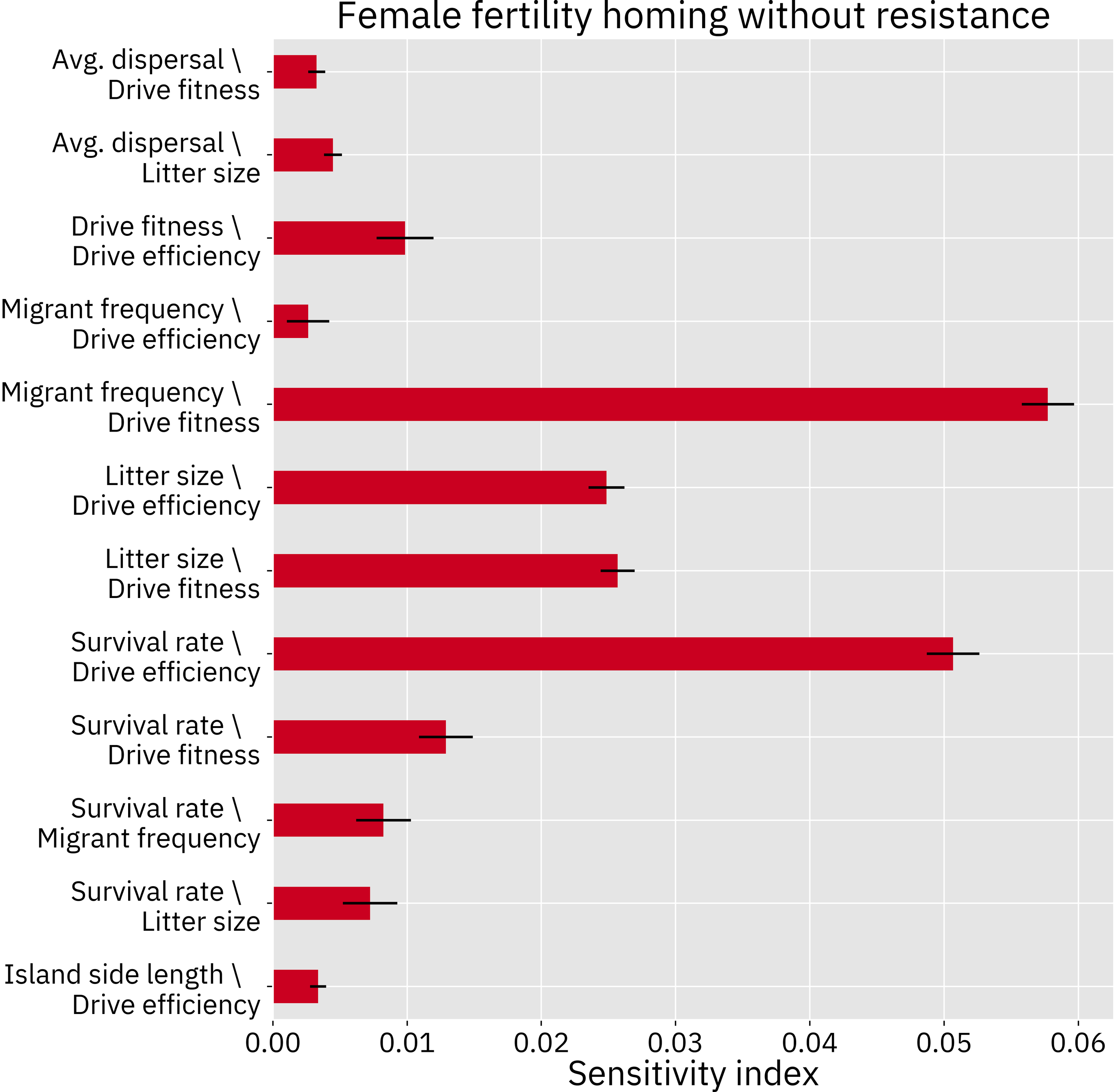

Supplement: S6 Fig — Second order effects describe the pairwise synergies of two parameters. Only the 12 largest effects are shown. (TIF) [file pcbi.1009660.s006.tif]

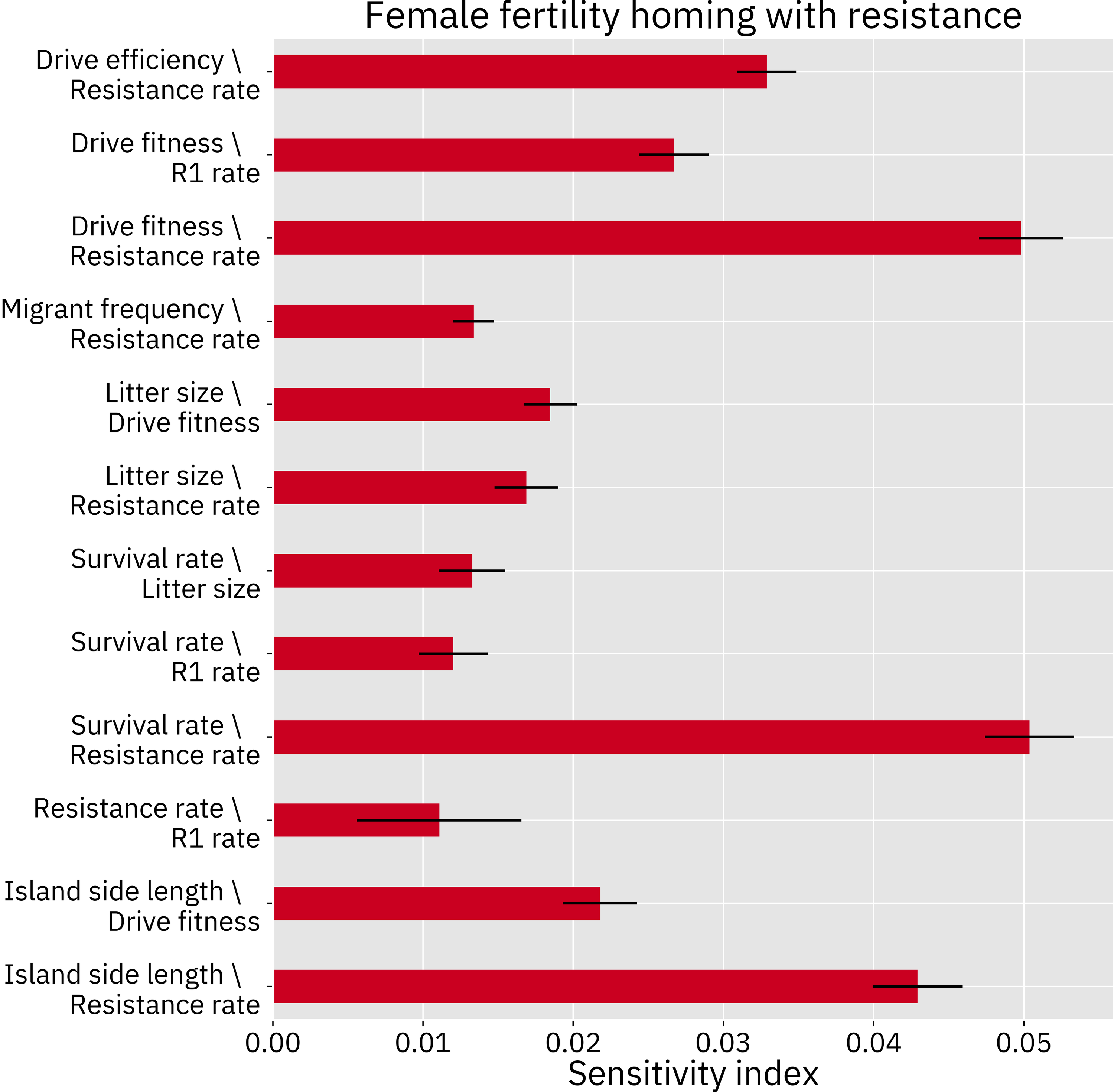

Supplement: S7 Fig — Second order effects describe the pairwise synergies of two parameters. Only the 12 largest effects are shown. (TIF) [file pcbi.1009660.s007.tif]

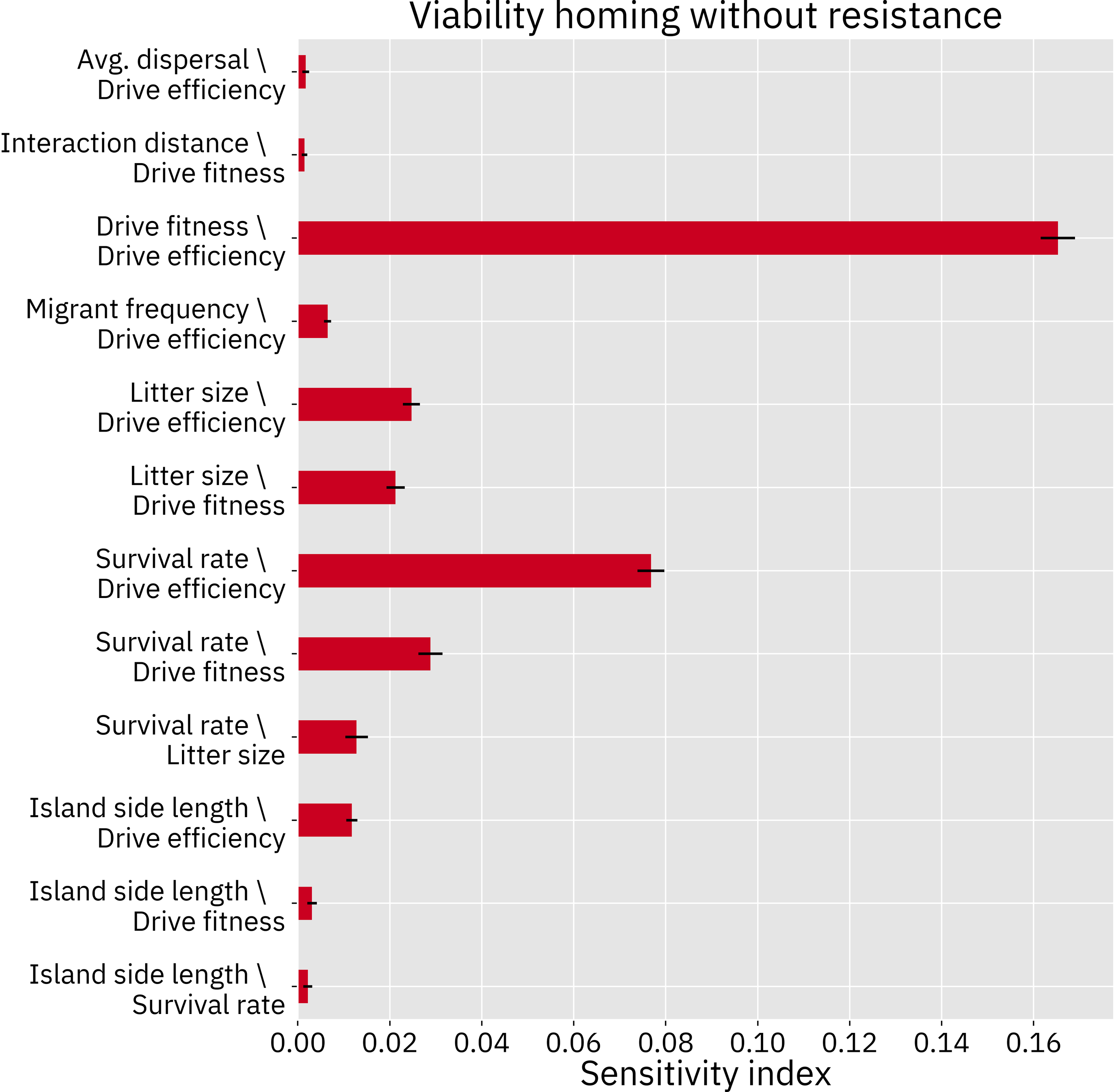

Supplement: S8 Fig — Second order effects describe the pairwise synergies of two parameters. Only the 12 largest effects are shown. (TIF) [file pcbi.1009660.s008.tif]

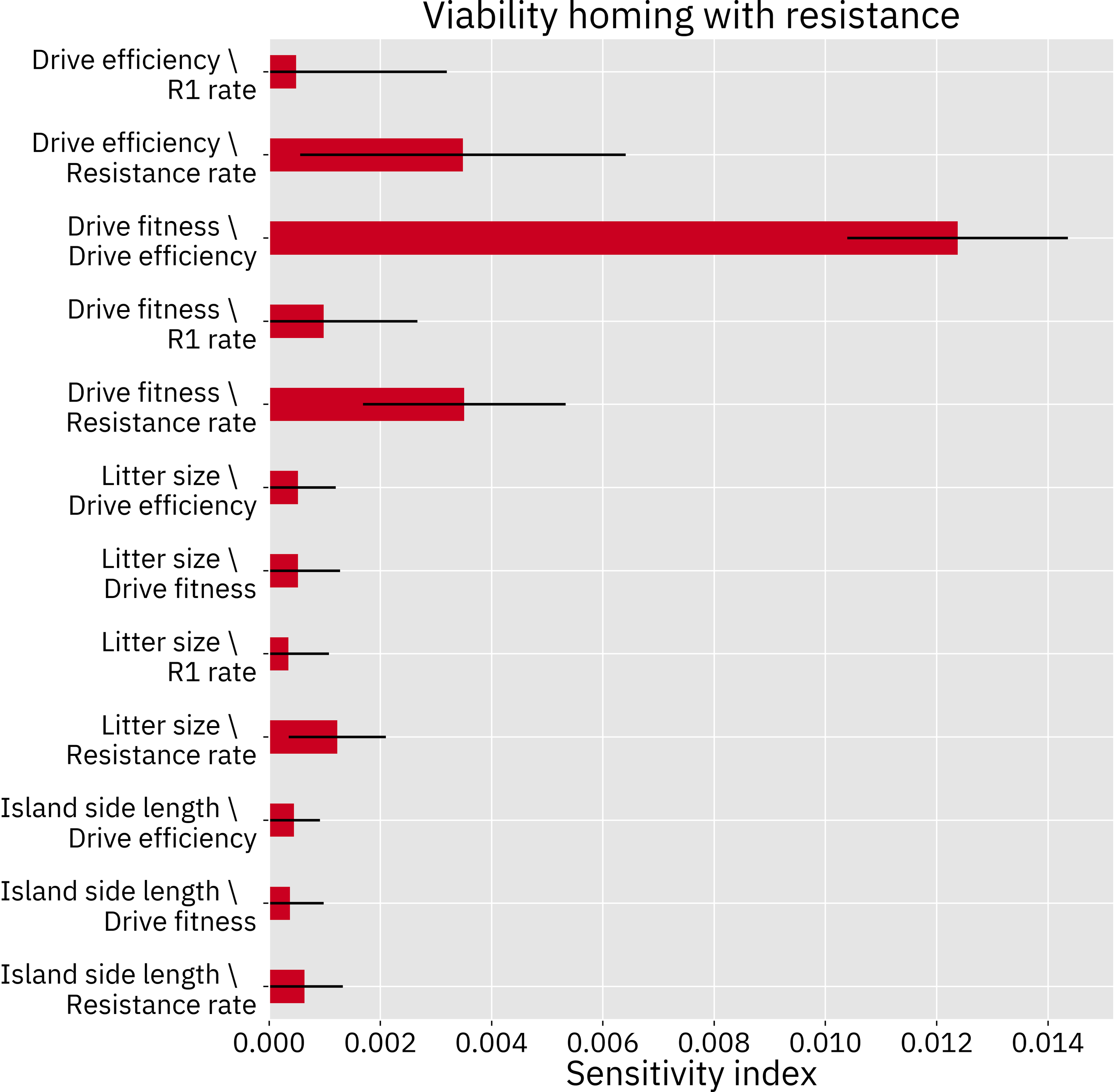

Supplement: S9 Fig — Second order effects describe the pairwise synergies of two parameters. Only the 12 largest effects are shown. (TIF) [file pcbi.1009660.s009.tif]

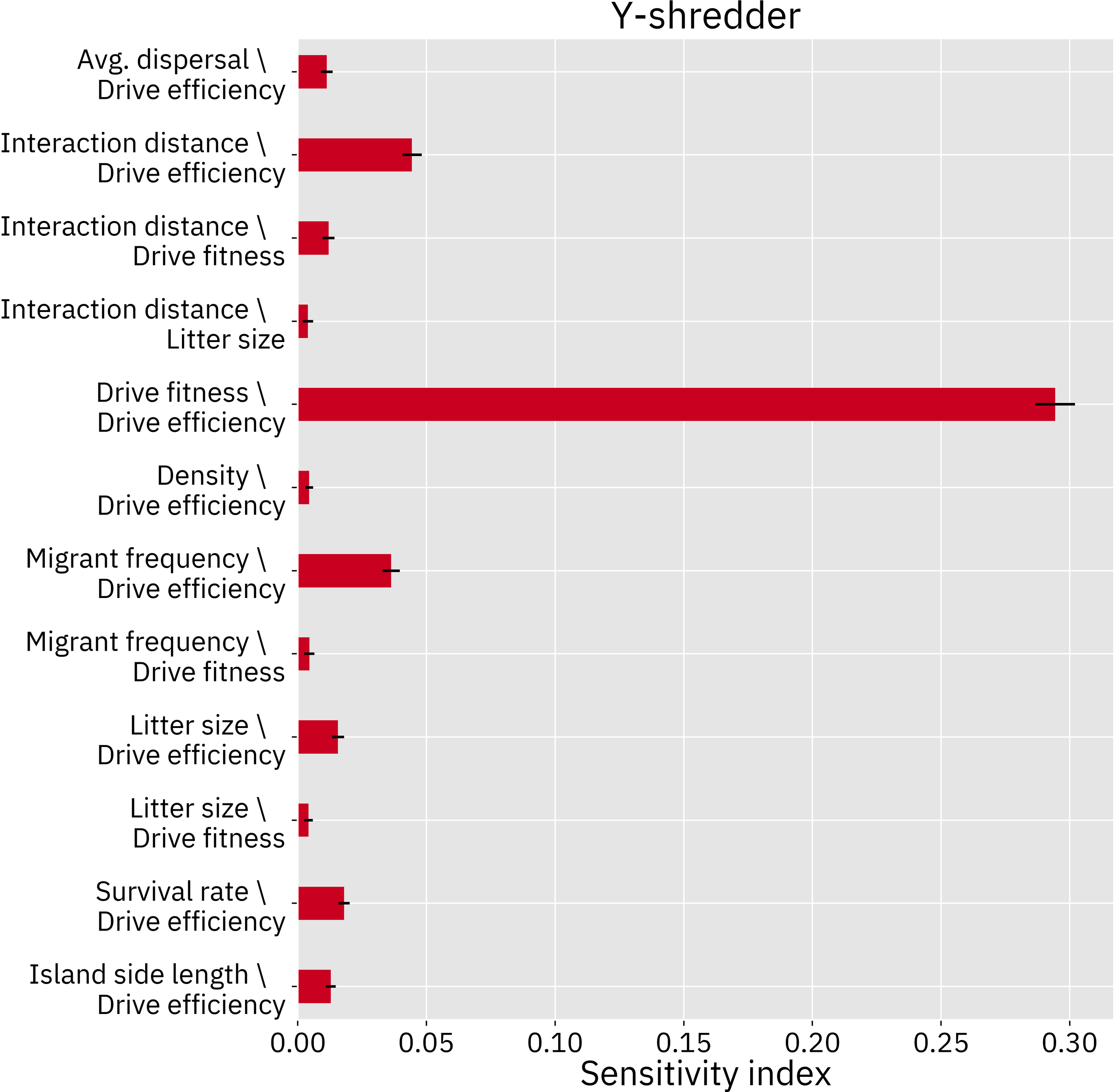

Supplement: S10 Fig — Second order effects describe the pairwise synergies of two parameters. Only the 12 largest effects are shown. (TIF) [file pcbi.1009660.s010.tif]

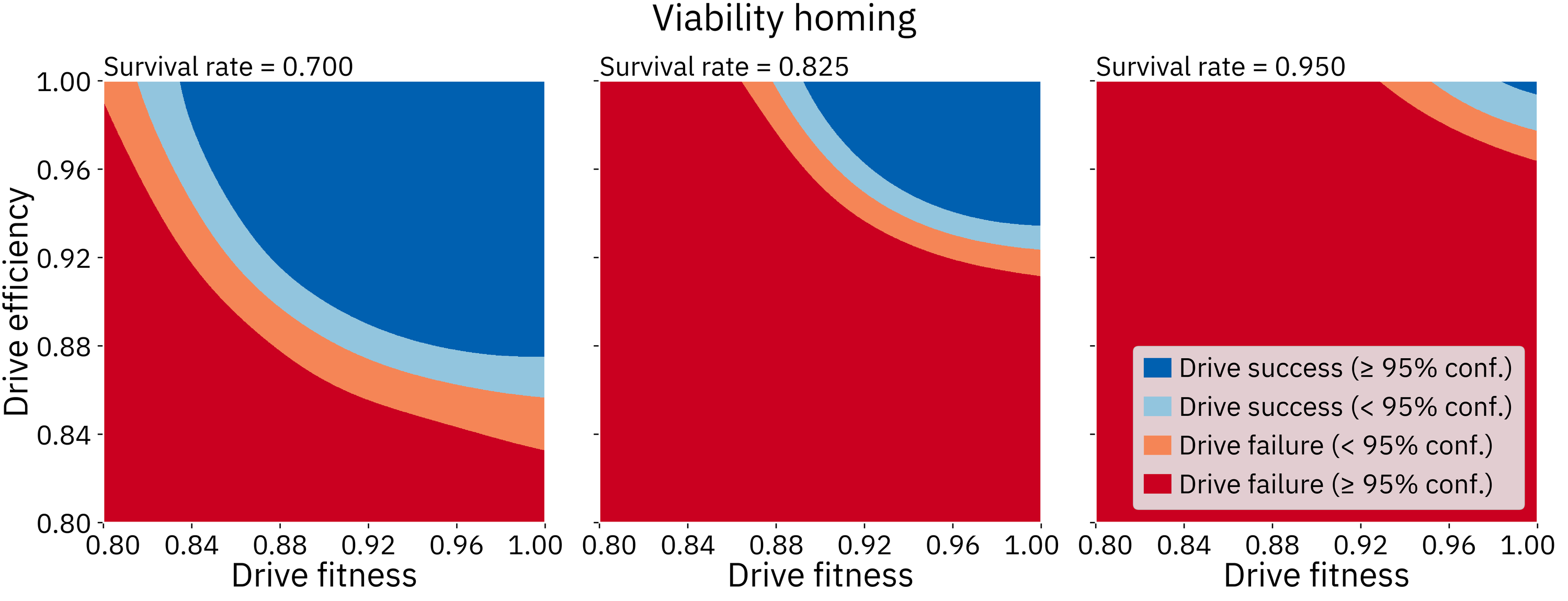

Supplement: S11 Fig — Other parameters are fixed at default values. An initial analysis (in Fig 11) suggested that litter size was not important for this drive; however, this analysis confirms that litter size can make the difference between success and failure of the drive, as indicated by sensitivity analyses (Figs 8 and 9). (TIF) [file pcbi.1009660.s011.tif]

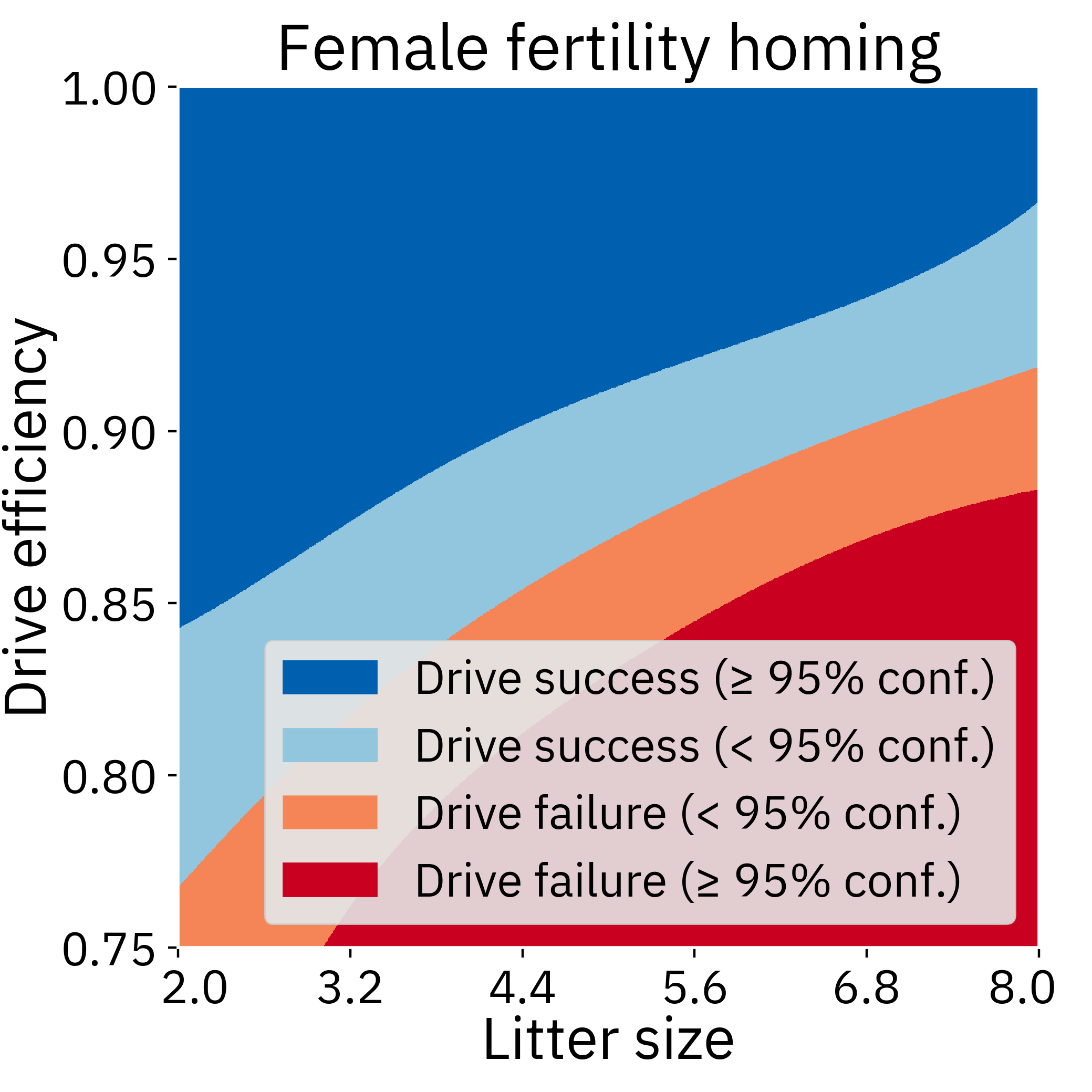

Supplement: S12 Fig — Other parameters are fixed at default values. (TIF) [file pcbi.1009660.s012.tif]
