# Supplementary material for: Modeling CRISPR gene drives for suppression of invasive rodents using a supervised machine learning framework
Source: PLoS Comput Biol. 2021 Dec 29;17(12):e1009660. doi: 10.1371/journal.pcbi.1009660 (PMC8716047; doi:10.1371/journal.pcbi.1009660)
Supplement: Attachment — Submitted filename: Review.pdf [file pcbi.1009660.s014.pdf]

# Peer review “Modeling CRISPR gene drives for suppression of invasive rodents”

## Summary and overall impression

This manuscript explores the entire parameter field of a rat gene drive population model using a Gaussian Process meta-model. The authors show that this method is computationally feasible, produces reliable results, and provides an excellent starting point for investigating certain parameter combinations specifically through an extensive sensitivity analysis.

This manuscript fits the scope of PLOS Computational Biology, as it uses a novel computational approach, a Gaussian Process meta-model, to elucidate the biological scenarios in which a gene drive is successful in a rodent population. The underlying rat model is slightly more detailed than previous rodent models. The authors test three types of gene drive and use two measures to analyse gene drive success in their model, which capture separate and important gene drive dynamics: the suppression rate and a “composite” value, which combines the speed of suppression with the magnitude of suppression.

The manuscript is clearly structured, well written, and has clear and intuitive figures.

This type of meta-model will be especially valuable for non-model species with unusual life histories, or where many ecological parameters are still unknown. Through this method, specific parameters can be pinpointed that are most impactful for gene drive success or failure, which can then be prioritised in field research.

I advise to publish this manuscript with minor revisions.

## Major and minor issues

The most important issue with this manuscript is that both the title and introduction focus on the rodent-aspect of this research, whereas the meta-model approach is the most significant novel aspect and focal point in this manuscript. I would advise to rewrite the title and the introduction to fit this aspect better.

Secondly, although the text on the GP model is clearly written (line 373-456), the overall picture of the model is lost in detail because there is so much going on. A schematic overview figure will be of big help here.

Besides these two points, several minor improvements can be made to increase the clarity of the manuscript.

- All figures have several panels, which, though clearly labelled, make referencing from the text somewhat difficult. I suggest labelling sub-figures (using a, b, c, etc.) for straightforward referencing in the text (for example line 513, 518, and 527 in the case of Figure 1)
- Great job on the separate mortality rates for migrants and new-borns, this has been a feature missing in most models to my knowledge. However, I wonder why the authors chose to use the word “itinerants” for migrating individuals? “Itinerant” is a word that might not be immediately understood by non-native English speakers, while the word “migrants” is clearer. Furthermore, “itinerant frequency” (line 223 and others) makes

it sound as though there is a certain fraction of individuals that live a migratory lifestyle, whereas what is meant is the migrating frequency in a certain timestep only. Perhaps, changing this term to "migrating frequency" will make this more intuitively clear.

- All figures in the manuscript are good-looking and intuitive to interpret. Although the colour scheme of all figures (mostly the heat maps) is good for both colour-seeing and most colour-blind people, in greyscale it is almost impossible to see the difference between the blue and red colours:

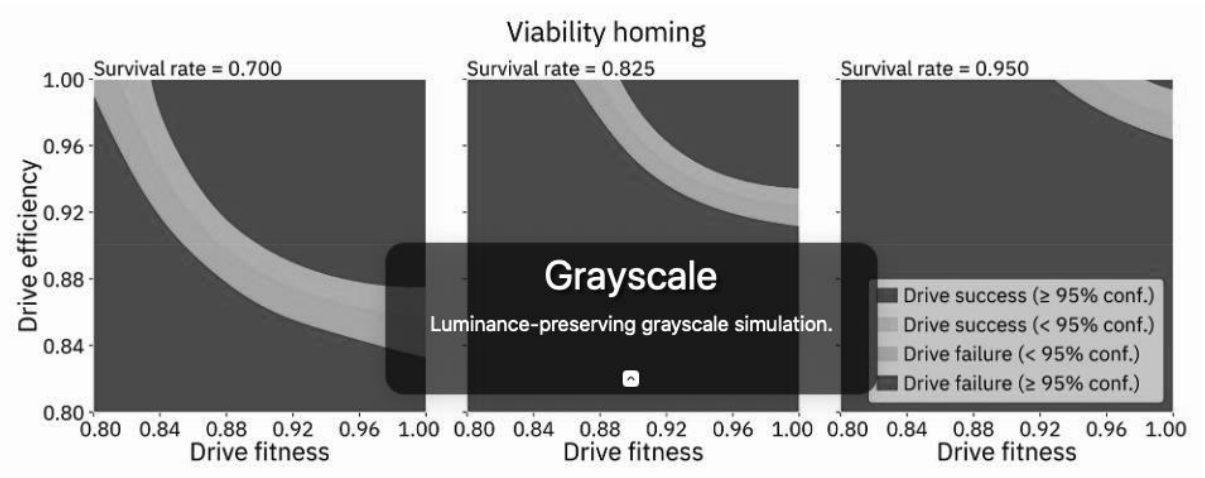


  This makes the figures inaccessible for people with poor vision, and also when the figures are printed in greyscale. I would suggest changing the saturation of either colour or adding hatching to them to make this figure more accessible. I use the app "color Oracle" to check figure accessibility.

- Figures 2 and 3 provide a clear visual in the narrative of the manuscript. However, since only one type of gene drive is plotted here, this causes some confusion later on. Supplemental figure 1 seems to be referenced in the text more than these two figures. Perhaps a figure that shows data for all three gene drive types might be a better choice here, either through adding this data in separate panels or by switching these two with supplemental figure 1. Another option is to plot the average timesteps until eradication for a nice comparison of gene drive types (line 548 and 561). Supplemental figure 1 should also be referenced in the text from line 534 to 556.
- Line 568-592 makes a great point on how to interpret gene drive "success" or "failure". I think these paragraphs are more suited for the discussion though.
- Figure 4 contains data to demonstrate the improvement in precision and recall after increasing amounts of training. However, this figure shows only the models of one gene drive type as a main figure, with the rest being in the supplementary. Although the figure is only used to illustrate that training improves the model (line 645), this does raise questions on why only the best performing model is shown as a main figure. Therefore, I would suggest splitting figure 4 into four panels (precision and recall, and suppression rate and composite value), and plot the models for all gene drives in the main figure. This way it is possible to compare model performance per gene drive and per model training step, while it's still clear that the models improve with training.
- Figure 5 are very nice graphs to directly compare model outcomes with GP predictions. However, as mentioned in line 660-663, the model without resistance has the highest model accuracy. In the context of investigating the merits of a GP meta-model, it is

fairer and more useful to also show a figure like this for a model with a lesser performance.

- Line 716: “For purposes of this set of analyses, models without resistance were used.” From this sentence, it is not clear to me why the models without resistance were used.
- In the section “Selected model outcomes” and Figure 8 and 11, it is not clear it the models include resistance or not until line 777.
- Line 803-813 include novel results (which are quite exciting!), so this paragraph is more suited to the results than the discussion.
- Line 878-879 suggest that the adaptive sampling could be improved by adaptively sampling points during training, but from line 427-456 I understand that the training was already adaptive. What specifically is meant here?
- In the conclusion or discussion, there is no mention of either rats or rodents. Although I suggested above to change the angle of the title and introduction, it would still be nice to discuss how this modelling work fits with previous work and current challenges in the rat/rodent invasiveness problem.
- Instead of “timestep” in the plots, “generation” would be a clearer description of what is actually happening. You mention that a timestep could be any amount of time from 1 to 6 months, but by using “generation”, this is immediately clear.
- Line 90-93 makes it sound as though the time steps in this model will be non-discrete and non-overlapping, which is confusing because this was not done (line 210).
- Line 227: typo, remove “as”
- Table 1: the maximum island side length used is 5 km, which is not terribly large, whereas the introduction specifies that this is a challenge (line 80-82). Would the computational feasibility of the GP model computationally hold up with a larger, if not huge, island size?
- In table 1 and table 2, I would like to have sources for the chosen parameters. Only 1000 m migration distance seems quite little for a rat based on intuition.
- In table 1, the term “survival rate” is not intuitively linked to the paragraph “mortality”, I would choose either term for consistency.
- Line 510-513 it is not clear what the “minor discrepancies” are, this needs more explaining.
- Line 411: Explain what “hyperparameters” are here.
- Line 373: A header “Training” instead of “Quality assessment” might be clearer, as the combination of “training and testing” is clearer than “quality assessment and testing”.
- Line 489-493: I would move this motivation for this type of sensitivity analysis to the top of the section.

All in all, with these improvements, this is a great manuscript with a valuable new gene drive modelling approach.
