# Supplementary material for: Modeling CRISPR gene drives for suppression of invasive rodents using a supervised machine learning framework
Source: PLoS Comput Biol. 2021 Dec 29;17(12):e1009660. doi: 10.1371/journal.pcbi.1009660 (PMC8716047; doi:10.1371/journal.pcbi.1009660)
Supplement: Attachment — Submitted filename: Response to Reviewers - Suppression of Invasive rodents.docx [file pcbi.1009660.s015.docx]

Dear Dr. Althouse,

Thank you for your consideration of our paper “Modeling CRISPR gene drives for suppression of invasive rodents using a supervised machine learning framework” and for providing the valuable feedback by the reviewers. Please find attached a new version of the manuscript, which we revised in response to their comments. Two instances of the manuscript are attached – one in which all changes from the original submission are tracked, along with a clean version. We hope that the revisions satisfactorily address all remaining concerns. Below we provide point-by-point responses to all specific comments.

Sincerely,

Samuel Champer and Philipp Messer (on behalf of all authors)

**REVIEWERS' COMMENTS:**

**Reviewer 1**

**Reviewer #1: In this article, Champer et al. have used an existing population genetic framework to simulate the impact of suppression gene drive strategies in eliminating rat species in an island setting. Given the large parameter space associated with individual-based dynamical systems they use a Gaussian process model to learn important parameters and their associated value ranges that would lead to gene drive success. They also perform sensitivity analyses on the model albeit the GP one. This article contains a number of details but I think the authors could do a better job of highlighting the take home points and the major contribution of their work. I am unclear if this work aims to evaluate target profiles of rat gene drives, how to develop an emulator, model a specific physical context, none of these, or all of these?**

We consider our paper to provide two major contributions. (i) a study of how a more realistic spatial model of rodent life cycle can affect the outcome of a suppression gene drive, and (ii) an introduction of the Gaussian process methodology as a tool for studying such a complex ecological system. We do not necessarily want to prioritize one of these contributions over the other (although different readers may personally find one or the other more interesting), and our conclusion paragraph gives equal space to both. As part of our changes to make this clearer, we added language to the introduction to highlight the Gaussian process portion, as this had previously been understated in the first part of the paper. We have also changed the title to “Modeling CRISPR gene drives for suppression of invasive rodents using a supervised machine learning framework”. This should signal to readers that the machine learning aspect of the paper is also part of its core purpose.

**The parameter space is large for these models, however, a number of these parameters ought to be tied to real world data (such as migration rates as well as gene drive parameters), which should limit the parameter space greatly. In fact, this has been proven multiple times with gene drive models of mosquitoes and other insects. So as a major suggestion, I request the authors to tighten up the language to communicate their goals and what they did to achieve them more clearly. Second, I would like to see more grounding in data. Rat migration and reproduction are well studied in literature and making assumptions about these characteristics without grounding them in data does not engender confidence in the reader.**

Our parameter ranges were chosen based on a systematic literature review one of our coauthors conducted for Manaaki Whenua, New Zealand’s Crown Research Institute for Landcare Research. We apologize that this was not made entirely clear in the original manuscript. We now mention this in more detail in the manuscript and also provide a citation to the report describing the methodology of the literature review and its results, which is freely available on Manaaki Whenua’s data repository website.

We intentionally kept the parameter ranges sufficiently broad to ensure that almost all real-world populations will fall somewhere within the possible parameter space. This includes populations of both Black and Brown rats – dense, urban populations as well as sparse, savanna populations. This goal is now more clearly stated in the methods section.

One nice feature of the machine learning technique is that is it quite adaptable to analyzing subsections of the parameter space. If the initial net is sufficiently wide, this methodology allows us to easily zoom in and predict results in a more narrowly defined population as well. Thus, it would be straightforward to significantly shrink the parameter ranges to fit values assessed in a given real-world population.

**More specific comments are as follows:

Introduction:**
 **1. In the section on spatially abstract models, the following two would be very relevant to include because they are models explicitly created to simulate genetics and gene drive models as opposed to spatial models of disease transmission that then included gene drive as an add on:

Sanchez et al. (2019):** [**https://besjournals.onlinelibrary.wiley.com/doi/full/10.1111/2041-210X.13318**](about:blank) **Selvaraj et al. (2020):**
[**https://journals.plos.org/ploscompbiol/article?id=10.1371/journal.pcbi.1008121**](about:blank)

We thank the reviewer for pointing us towards these papers, which are great examples for supporting the proposition that spatial structure has an important impact on the predicted success or failure of a gene drive. We have added these citations to the introduction section.

**Methods:
1. "Time steps in the simulation are equivalent to one breeding cycle, which in reality can range from approximately one to six months depending on the availability of resources, as well as the climate and time of year" Please mention how this compares to the average lifespan of the species being modeled.**

Lifespan is now mentioned in the methods section. We have also included the average age and average life expectancy of the model with default parameters in the results section.

**2. "The simulation is then run for an additional 500 time steps or until the population is eliminated." Does the 500 time steps imply there are scenarios where elimination is not achieved?**

Yes – there is a very small set of simulations where suppression would likely have been achieved had the simulation been run for another 100 time steps. This number of additional suppressions (if sampling randomly from the parameter space) is perhaps around 1%. The chosen limit of 500 time steps is of course somewhat arbitrary, but was selected as a very permissive upper bound for the gene drive (it would correspond to ~80 years when assuming a breeding cycle of 2 months). This is now clarified in the manuscript.

**3. Why was a Gaussian curve chosen for competition exerted? Is this fit to some data showing loss in attraction or purely an assumption? Please clarify.**

A Gaussian competition kernel is a common assumption in many ecological models (see, e.g., Doebeli and Dieckmann, 2003: https://doi.org/10.1038/nature01274) and is an assumption in our model as well. That said, it is an assumption that fits data in the sense that wild populations of rats are not hyper-clustered on top of one another. In any case, we have added a citation supporting the simulation of competition in this manner.

**4. Are there other fitness costs apart from fertility and viability?**

Fitness in the model only directly effects viability (of course, fecundity is impacted in the obvious sense that individuals that perish might have otherwise been able produce additional litters had they survived). This has been clarified in the text. Note that these fitness effects are separate from the intended effects of the drive (such as sterility in female drive homozygotes in the female fertility homing drive).

**Gaussian Process:
1. Are 20 replicates at each simulated point enough to estimate stochastic variation in the model?**

Yes. Twenty replicates is more conservative than we needed to be. Our models would have lost little accuracy if that number were as much as halved. The model produces stochastic outputs (e.g., precisely how long did it take for the population to be suppressed). However, due to the abrupt transition between drive success and drive failure, very few simulated points had meaningful variation from simulation to simulation in this regard. For those rare points where such variation exists, we don’t need to know the exact frequency at which the drive succeeds or fails: just the fact that we see that variation gives the Gaussian process plenty of useful information about the model. Given a point with a variable outcome, we know that the drive will succeed if we move through the space just a little bit in a direction favorable to the drive (such as an increase to drive efficiency) and will fail if we move in a direction unfavorable to the drive.

**2. "This value is composed of two terms: one denoting the speed at which suppression occurred (if it occurred) consisting of the last generation in which there were living individuals divided by the total number of generations simulated (500)" - Isn't this true for the denominator only if the simulations haven't eliminated before 500 steps?**

We agree that the equations as presented were not as clear as they could be. We have reworked this section to clarify the method.

**3. If the suppression model can produce biased results because of sharp transitions, why use the suppression model at all? Isn't it better to just stick to the composite model?**

If we had ideal methods, just using one Gaussian process method would have been preferable for the sake of clarity and simplicity. However, until we have sufficiently refined our methods, both models provide value – the composite model is better in some ways (and was especially useful before adaptive sampling improved the data sets), while the “suppression rate” model performs better in other ways (after all, predicting whether the drive will suppress or not is precisely the outcome we are seeking to predict). **Results:
Population Model:
1. What exactly is the discrepancy in the density calibration model? This feels a bit hand wavy and I'd appreciate more detail here because the point of an agent based model is to be able to track individuals explicitly and not having exact control over the population size does not make sense to me.**

Our model has a “density” parameter (which is explicitly controlled), but this parameter does not explicitly set the number of individuals in the model. Rather, the density (along with several other parameters) is used to determine a “density tuning coefficient” that in turn results in the system having approximately the desired population size. However, this calculation is only an approximation of the model itself and doesn’t take into account variations in density that may occur across the landscape, individuals failing to find mates, or any stochastic factors.

Thus, the exact population density can vary by a small amount from the amount specified. We have added some text to the results section to clarify this. **2."This correlates fairly well with the default maximum competition distance of 75 meters". Is this some standard distance for max competition?**

Nothing is special about 75 - this is just the default interaction distance parameter we chose for the model. We have clarified the text to indicate this.

**3."The transition between invariable failure and invariable success occurs rather abruptly as efficiency is increased beyond a threshold". Is this a feature of model dynamics? Why does this happen? The authors addressed how they got around this while using the Gaussian Process model but I still don't understand why this is happening.**

This is a phenomenon that is seen in a lot of gene drive modeling and is not unique to our rat model. A drive that meets a certain efficiency threshold (which is “good enough” for a particular scenario) will almost always spread through and then suppress a population. A drive that falls short of that threshold will reach an equilibrium or be lost. A drive on the borderline might succeed half the time, but if such a drive had even 1% more or less efficiency, the drive would the outcome would be much more certain.

An extremely noisy model, such as one with a very small population size, might exhibit blurrier thresholds.

**4. In figure 2, I see there are simulations that eliminated at 490 time steps. Are there simulations that would have eliminated at 510 time steps? Why is 500 chosen as the maximum simulation time? The definition for elimination/persistence has to be more rigorous than an arbitrarily chosen time step.**

We chose 500 time steps as a duration that is highly permissive to the gene drive, and which would allow almost every drive that could possibly succeed to reach success. This is a somewhat arbitrary choice; however, the goalposts that will be used to measure any potential release candidate gene drive are similarly arbitrary. A regulatory entity contemplating a drive release will need to decide about what the drive must accomplish. Perhaps a requirement for a drive will be that a population be eliminated within a decade, or perhaps within 50 years – this is not a decision that will be made by the engineers of a drive, yet it will determine which drives are considered candidates and which are considered insufficient.

**5. Where exactly are the genetically modified rats released? Figure 3 suggests a random release?**

That is correct. The methods section states that the individuals are randomly scattered across the landscape. Drive release strategy is possibly very important, depending on the drive type, and will warrant substantial consideration before any real-world release. However, for this study, we chose to keep the release simple.

**6. I'm still confused about the two GP models. In the methods, the authors state the composite model would offer a solution around abrupt transitions but it looks like the accuracy is a lot lower than the suppression only GP model. So what is the way to go then?**

Both models have strengths and weaknesses. The composite model tended to exhibit stronger accuracy before adaptive sampling (except when resistance was simulated). After adaptive sampling, the “suppression rate” GP scored better accuracy. However, the accuracy numbers don’t quite capture everything about the models. We have added further discussion of the differences between the two models to make it clearer what the individual strengths of each model are and why we feel it is useful to keep both models.

**7. "it appears that the suppression rate model often has areas of more exaggerated curvature than the composite model". Is this with reference to figure 5? I'm not sure I see the exaggerated curvature. What do the authors mean here?**

We have removed this confusing sentence and have added a more in-depth paragraph discussing the differences between the models.

**Reviewer 2**

**Summary and overall impression**

**This manuscript explores the entire parameter field of a rat gene drive population model using a Gaussian Process meta-model. The authors show that this method is computationally feasible, produces reliable results, and provides an excellent starting point for investigating certain parameter combinations specifically through an extensive sensitivity analysis.**

**This manuscript fits the scope of PLOS Computational Biology, as it uses a novel computational approach, a Gaussian Process meta-model, to elucidate the biological scenarios in which a gene drive is successful in a rodent population. The underlying rat model is slightly more detailed than previous rodent models. The authors test three types of gene drive and use two measures to analyse gene drive success in their model, which capture separate and important gene drive dynamics: the suppression rate and a “composite” value, which combines the speed of suppression with the magnitude of suppression.**

**The manuscript is clearly structured, well written, and has clear and intuitive figures.**

**This type of meta-model will be especially valuable for non-model species with unusual life histories, or where many ecological parameters are still unknown. Through this method, specific parameters can be pinpointed that are most impactful for gene drive success or failure, which can then be prioritised in field research.**

We thank the reviewer for these kind comments.

**I advise to publish this manuscript with minor revisions.**

**Major and minor issues**

**The most important issue with this manuscript is that both the title and introduction focus on the rodent-aspect of this research, whereas the meta-model approach is the most significant novel aspect and focal point in this manuscript. I would advise to rewrite the title and the introduction to fit this aspect better.**

We agree – the meta-model aspect of the research is something we are very excited about. We have suggested a change to the title of the paper, which now reads: “Modeling CRISPR gene drives for suppression of invasive rodents using a supervised machine learning framework”. While we initially considered opening the introduction with the machine learning section, we found that there was no good way to introduce our solution without first introducing our problem, so to speak. However, we have added emphasis to the machine learning elements in the introduction.

**Secondly, although the text on the GP model is clearly written (line 373-456), the overall picture of the model is lost in detail because there is so much going on. A schematic overview figure will be of big help here.**

We thank the reviewer for this excellent suggestion and have added a schematic figure. We agree that it substantially increases the readability of this section.

**Besides these two points, several minor improvements can be made to increase the clarity of the manuscript.**

**• All figures have several panels, which, though clearly labelled, make referencing from the text somewhat difficult. I suggest labelling sub-figures (using a, b, c, etc.) for straightforward referencing in the text (for example line 513, 518, and 527 in the case of Figure 1)**

Adding letter sub-labels to the figures would have the unintended result of squashing the current labels in some of the figures (especially figure 6, wherein labels are already heavily abbreviated). However, we looked through our references to the figures throughout the paper, and noticed that figure 2 is the only figure where we reference the panels separately. Our references now clarify which panel is being referenced by specifying “left panel”, “center panel”, or “right panel”.

**• Great job on the separate mortality rates for migrants and new-borns, this has been a feature missing in most models to my knowledge. However, I wonder why the authors chose to use the word “itinerants” for migrating individuals? “Itinerant” is a word that might not be immediately understood by non-native English speakers, while the word “migrants” is clearer. Furthermore, “itinerant frequency” (line 223 and others) makes it sound as though there is a certain fraction of individuals that live a migratory lifestyle, whereas what is meant is the migrating frequency in a certain timestep only. Perhaps, changing this term to “migrating frequency” will make this more intuitively clear.**

We have made the change from “itinerant” to “migrant” throughout the manuscript to increase clarity, as suggested.

**• All figures in the manuscript are good-looking and intuitive to interpret. Although the colour scheme of all figures (mostly the heat maps) is good for both colour-seeing and most colour-blind people, in greyscale it is almost impossible to see the difference between the blue and red colours.**

**This makes the figures inaccessible for people with poor vision, and also when the figures are printed in greyscale. I would suggest changing the saturation of either colour or adding hatching to them to make this figure more accessible. I use the app “color Oracle” to check figure accessibility.**

We have implemented several changes to the figures that should help with greyscale without reducing the visual appeal or the color-blind friendly nature of the figures.

The color scheme has been changed in all heatmap figures (formerly fig 5, 8, 9, 10, 11, 12, sup 8, sup 9 [all figure numbers are now changed because of the addition of the schematic figure and because supplemental figure one has been moved to the main text]). The light-red color is now a darker orange color. This makes it possible to distinguish between light red areas and light blue areas even in greyscale. The dark blue and dark red colors in these figures are still somewhat similar in greyscale, but those two colors are never present next to one another, so there is no danger of these colors blending together (and it should usually be obvious which area of the graph is drive success and which is drive failure).

The color scheme in the model comparison figures (formerly fig 5; there are now four new model comparison figures for the other models) has also been changed: the simulated points which were overlaid on the Gaussian process results have been changed for increased clarity (especially when printed in greyscale).

The color scheme of all line graph figures (former fig 4, sup1, sup2) and figure 2 has also been changed. These figures previously used dark red and dark blue, which are hard to tell apart in greyscale. The dark red color has been changed to a less saturated orange color.

**• Figures 2 and 3 provide a clear visual in the narrative of the manuscript. However, since only one type of gene drive is plotted here, this causes some confusion later on. Supplemental figure 1 seems to be referenced in the text more than these two figures. Perhaps a figure that shows data for all three gene drive types might be a better choice here, either through adding this data in separate panels or by switching these two with supplemental figure 1. Another option is to plot the average timesteps until eradication for a nice comparison of gene drive types(line 548 and 561). Supplemental figure 1 should also be referenced in the text from line 534 to 556.**

Supplemental figure 1 has been moved to the main text.

As for figures 2 and 3 only showing one drive: these figures are only intended to show the general trend of the expected dynamics of a release and are not intended as exhaustive analyses. Similar trends are exhibited by the other drives at different efficiency levels.

**• Line 568-592 makes a great point on how to interpret gene drive “success” or “failure”. I think these paragraphs are more suited for the discussion though.**

We agree that these paragraphs do not belong in the results section. While the section is discussion-like in tone, the paragraphs also serve to explain what our model is measuring. Clarifying this before introducing our Gaussian process methodology actually makes the most sense to us. Therefore, we have merged these paragraphs together, tightened the wording, and moved the paragraph to the methods section.

**• Figure 4 contains data to demonstrate the improvement in precision and recall after increasing amounts of training. However, this figure shows only the models of one gene drive type as a main figure, with the rest being in the supplementary. Although the figure is only used to illustrate that training improves the model (line 645), this does raise questions on why only the best performing model is shown as a main figure. Therefore, I would suggest splitting figure 4 into four panels (precision and recall, and suppression rate and composite value), and plot the models for all gene drives in the main figure. This way it is possible to compare model performance per gene drive and per model training step, while it’s still clear that the models improve with training.**

We found there was no way to fit every precision/recall panel on a single page without substantially decreasing readability.

The female fertility targeting gene drive was our “default drive” from the outset of the study, and we believe that this is an appropriate choice for a few reasons. For many in the gene drive field, this is the most relevant drive of the three we present. This type of drive has already been extensively analyzed in other studies and has even successfully eliminated cage populations of mosquitoes.

By contrast, the drive that renders homozygous embryos non-viable is probably less relevant. This drive is present in our study as a result of our group stepping back and saying to ourselves: “what is the most straight forward possible homing suppression drive”. While we think the inclusion of this drive in our study is useful for this reason, as well as for comparison purposes, this is probably not a drive that anyone plans on engineering (which, our results indicate, is probably reasonable).

Similarly, we believe that the Y-shredder serves as a useful comparison and demonstrates some interesting properties of a non-homing gene drive (e.g., the relatively increased sensitivity of the drive to certain spatial factors). However, we don’t think there is as much interest in a non-homing Y-shredder in general as there is in the default female fertility drive. For this reason, we put the graph for the default drive in the body of the text and the rest in the supplement.

**• Figure 5 are very nice graphs to directly compare model outcomes with GP predictions. However, as mentioned in line 660-663, the model without resistance has the highest model accuracy. In the context of investigating the merits of a GP meta-model, it is fairer and more useful to also show a figure like this for a model with a lesser performance.**

As mentioned in the previous response, we believe that we are showing the results that will be of the broadest general interest.

However, unlike the precision/recall graphs, this figure did not have an equivalent version for the other models, and we agree that showing these additional comparisons would provide a more complete understanding of our results. Thus, equivalent figures for the other four Gaussian process models have been added to the supplement.

**• Line 716: “For purposes of this set of analyses, models without resistance were used.” From this sentence, it is not clear to me why the models without resistance were used.**

The models trained without resistance were selected due to their superior accuracy, as measured in the previous section of the results. This has now been clarified in the text.

**• In the section “Selected model outcomes” and Figure 8 and 11, it is not clear it the models include resistance or not until line 777.**

This is now specified at the beginning of that section of the results.

**• Line 803-813 include novel results (which are quite exciting!), so this paragraph is more suited to the results than the discussion.**

This is a something that we are quite excited about as well, but we decided that it would be very confusing to the flow of the paper if we moved this to the results section.

This is something we only thought to test after we completely finished the rest of our study, and only because the models themselves indicated that it might be interesting, and we do not consider this finding something that was as robustly assessed as the rest of our findings. Further, if we put this finding in the results, it would only make sense to add it to the methods as well. But since the parameter was not initially part of the model, we would have to explain why one single parameter is not included in the Gaussian process portion of the method, along with a post-hoc explanation of why the parameter is present. We think this would be unduly confusing, so we left it as a part of the discussion. However, we anticipate that this will be part of our modeling in future studies!

**• Line 878-879 suggest that the adaptive sampling could be improved by adaptively sampling points during training, but from line 427-456 I understand that the training was already adaptive. What specifically is meant here?**

The reviewer is correct that the models already used adaptive sampling. What we are referring to in the paragraph, is the methodology used to conduct our adaptive sampling. This methodology is something that we will aim to improve in future studies. This has been clarified in the text.

**• In the conclusion or discussion, there is no mention of either rats or rodents. Although I suggested above to change the angle of the title and introduction, it would still be nice to discuss how this modelling work fits with previous work and current challenges in the rat/rodent invasiveness problem.**

The first part of the discussion has several observations specific to the rat population aspect of the paper (before the following parts of the discussion center more on the computational methods). The conclusion also turns back to the rodent population.

**• Instead of “timestep” in the plots, “generation” would be a clearer description of what is actually happening. You mention that a timestep could be any amount of time from 1 to 6 months, but by using “generation”, this is immediately clear.**

We believe that calling our time steps “generations” would suggest to some readers that our population consisted of non-overlapping generations, as this is the norm in population modeling. We chose “time step” to avoid this confusion. We have added an extended description of what a time step refers to in our simulation models.

**• Line 90-93 makes it sound as though the time steps in this model will be non-discrete and non-overlapping, which is confusing because this was not done (line 210).**

Our model has discrete timesteps but overlapping generations. Line 210 was a comment along the lines of, “if this *were* a non-overlapping model, setting the population capacity would be straight forward, but since our model has overlapping generations, we did the following…”. However, this commentary clearly did more to confuse than it did to clarify, and has thus been removed, along with a retooling of the paragraph.

**• Line 227: typo, remove “as”.**

This line has been clarified.

**• Table 1: the maximum island side length used is 5 km, which is not terribly large, whereas the introduction specifies that this is a challenge (line 80-82). Would the computational feasibility of the GP model computationally hold up with a larger, if not huge, island size?**

The size limitation is entirely a product of the underlying model. A 5*5 island with the maximum allowed density within our parameter space (1500individuals/km^2) has a capacity of 37,500 adults. However, since newborns are fully simulated (and have spatial interactions with other individuals), this means, with the maximum litter size of 8, the model needs to do spatial calculations with up to 187,500 interacting individuals. We could simulate more individuals or larger areas if we make some compromises in other areas of the model.

The Gaussian process side of the method could be fed with data from any model – regardless of how computationally difficult the underlying model. While the Gaussian process method is not without limitations, we did not get near those limitations within the course of this study.

**• In table 1 and table 2, I would like to have sources for the chosen parameters. Only 1000 m migration distance seems quite little for a rat based on intuition.**

As per our response to reviewer one – we chose parameter ranges based on a systematic literature review conducted by one of our coauthors, which is now cited by the paper. Rats are definitely able to move further distances than 1km, and this is represented in our paper. The dispersal distance is used as the average for the exponential distribution for the displacement of newborns from their mothers, meaning that many rats indeed disperse well beyond 1 km.

Additionally, adult migrants have their movement multiplied by an “adult dispersal distance multiplier” parameter (formerly called “itinerant dispersal multiplier”) which ranges from one to five. The exponential distribution means that some individuals move substantially further. Considering that our maximum island size is a 5 km square, having a higher maximum displacement would have fairly little additional effect.

**• In table 1, the term “survival rate” is not intuitively linked to the paragraph.**

**“mortality”, I would choose either term for consistency.**

We have changed the paragraph heading to “survival rate” for consistency.

**• Line 510-513 it is not clear what the “minor discrepancies” are, this needs more explaining.**

This section has been clarified (and see the response to reviewer one above regarding the recurrence relation used to calculate the density tuning value).

**• Line 411: Explain what “hyperparameters” are here.**

Different Gaussian process co-variance functions, e.g., the Matérn kernel which we used in the manuscript, have varying numbers of hyperparameters that further determine model behavior. This very quickly gets into fairly heavy math, and the use of the term in the paper was distracting. This line has now been reworded.

**• Line 373: A header “Training” instead of “Quality assessment” might be clearer, as the combination of “training and testing” is clearer than “quality assessment and testing”.**

We have changed the header to “GP assessment”, as we believe this is the clearest option.

**• Line 489-493: I would move this motivation for this type of sensitivity analysis to the top of the section.**

We considered moving this paragraph, but the paragraph doesn’t make as much sense without the discussion in the preceding paragraph of how to choose the size of the “base sample” *n*.

**All in all, with these improvements, this is a great manuscript with a valuable new gene drive modelling approach.**

We again thank the reviewer for their kind comments.
